# Knowledge-based, computerized, patient clinical decision support system for perioperative pain, nausea and constipation management: a clinical feasibility study

**DOI:** 10.1007/s10877-024-01148-z

**Published:** 2024-04-12

**Authors:** Eric Noll, Melanie Noll-Burgin, François Bonnomet, Aurelie Reiter-Schatz, Benedicte Gourieux, Elliott Bennett-Guerrero, Thibaut Goetsch, Nicolas Meyer, Julien Pottecher

**Affiliations:** 1 Department of Anesthesiology, Intensive Care and Perioperative Medicine, Hautepierre Hospital, Strasbourg University Hospitals, Strasbourg, France; 2 Department of Pharmacy, Groupe Hospitalier Saint Vincent, Strasbourg, France; 3 Department of Orthopedic and Trauma Surgery, Hautepierre Hospital, Strasbourg University Hospitals, Strasbourg, France; 4 Department of Pharmacy, Hautepierre Hospital, Strasbourg University Hospitals, Strasbourg, France; 5 Department of Anesthesiology, Stony-Brook Medicine, Stony-Brook, NY USA; 6 Department of Biostatistics, Strasbourg University Hospitals, Strasbourg, France; 7 Department of Anesthesiology and Intensive Care, Hautepierre Hospital, Strasbourg University Hospitals, Strasbourg, France

**Keywords:** Perioperative medicine, Postoperative pain, Postoperative nausea and vomiting, Clinical decision support system, Pain management

## Abstract

Opioid administration is particularly challenging in the perioperative period. Computerized-based Clinical Decision Support Systems (CDSS) are a promising innovation that might improve perioperative pain control. We report the development and feasibility validation of a knowledge-based CDSS aiming at optimizing the management of perioperative pain, postoperative nausea and vomiting (PONV), and laxative medications. This novel CDSS uses patient adaptive testing through a smartphone display, literature-based rules, and individual medical prescriptions to produce direct medical advice for the patient user. Our objective was to test the feasibility of the clinical use of our CDSS in the perioperative setting. This was a prospective single arm, single center, cohort study conducted in Strasbourg University Hospital. The primary outcome was the agreement between the recommendation provided by the experimental device and the recommendation provided by study personnel who interpreted the same care algorithm (control). Thirty-seven patients were included in the study of which 30 (81%) used the experimental device. Agreement between these two care recommendations (computer driven vs. clinician driven) was observed in 51 out 54 uses of the device (94.2% [95% CI 85.9–98.4%]). The agreement level had a probability of 86.6% to exceed the 90% clinically relevant agreement threshold. The knowledge-based, patient CDSS we developed was feasible at providing recommendations for the treatment of pain, PONV and constipation in a perioperative clinical setting.

*Trial registration number & date* The study protocol was registered in ClinicalTrial.gov before enrollment began (NCT05707247 on January 26th, 2023).

**Supplementary Information:**

The online version contains supplementary material available at 10.1007/s10877-024-01148-z.

## Introduction

Postoperative pain control remains a major health issue [1, 2]. Opioids have typically been the cornerstone of postoperative pain management due to their strong analgesic properties [3]. Their indiscriminate use, however, is associated with potential major adverse effects [4]. Multimodal analgesia and patient-controlled opioid administrations are recommended strategies in the perioperative period to decrease opioid-related adverse effects [5, 6]. Oral, patient-controlled, opioid administration is particularly challenging in the perioperative context due to potential interactions between opioid administration and treatment of adverse effects from opioids, e.g. postoperative nausea and vomiting (PONV) and constipation.

Computerized-based Clinical Decision Support Systems (CDSS) are a promising innovation that might improve perioperative pain control. CDSS aim at enhancing medical decisions based on software-driven algorithms that take into account both clinical and patient information [7]. Development of CDSS can involve patient safety, clinical management or diagnostics support [8–10]. Patient decision support, administered directly to patients, is another potential development for CDSS [7, 11]. We believe that this technology may help patients and healthcare professionals to optimize medication management after surgery, particularly related to pain, nausea/vomiting (PONV) and constipation. We developed a knowledge-based CDSS aiming at making recommendations regarding the administration of perioperative pain, PONV and laxative medications as part of the Intelligent and Safe MEDication dispenser (InSAMED) project. This CDSS uses patient adaptive testing through a smartphone display, literature-based rules and individual medical prescriptions and produces direct medical advice for the patient user.

Our objective was to test the feasibility of the use by patients of our CDSS in the perioperative setting. We hypothesized that this knowledge-based CDSS would provide recommendations in agreement with clinician interpretation of the same algorithm (control).

## Method

### Trial design

This is a prospective single arm, single center, cohort study conducted in Strasbourg University Hospital from February 2023 to June 2023. The study protocol was approved by the institutional review board (chairman Pr Marie-France MAMZER-BRUNEEL, approval # 2022-A01926-37 on the January 9th, 2023). Written informed consent was obtained for every subject. The study protocol was registered in ClinicalTrial.gov before enrollment began (NCT05707247 on January 26th, 2023). The study was designed and conducted accordingly to ethical standards as reported in the Helsinki declaration. The development of the experimental device and the clinical study were funded by the SATT Conectus (Strasbourg University technology transfer office) and the Strasbourg University Hospitals. The data were collected in an electronical case-report form (cleanweb™).

This manuscript was constructed according to recommendations from both the STAtement on the Reporting of Evaluation studies in Health Informatics (STARE-HI) [12] and the CONsolidated Standards of Reporting Trials-Artificial Intelligence (CONSORT-AI) [13].

### Participants

Eligibility criteria included adults at least 18 years of age, planned surgery within a surgical department of the Hautepierre University Hospitals of Strasbourg, ability of the patient to understand and read French, convenience to interact neurosensorially with a tactile electronical interface, ability to understand research objectives, risks and provide dated and signed informed consent. Patients also had to be covered by health insurance. Exclusion criteria included neuropsychiatric or sensory disorders that could interfere with their use of the visual interface, inability to provide reliable symptom information, pregnancy or lactation, and subjects under safeguard of justice, guardianship or curatorship. Eligible patients were approached in the preoperative period in the surgical department. We prioritized surgical procedures involving of hospital length of at least 2 days.

### Experimental device

**Fig. 1:**
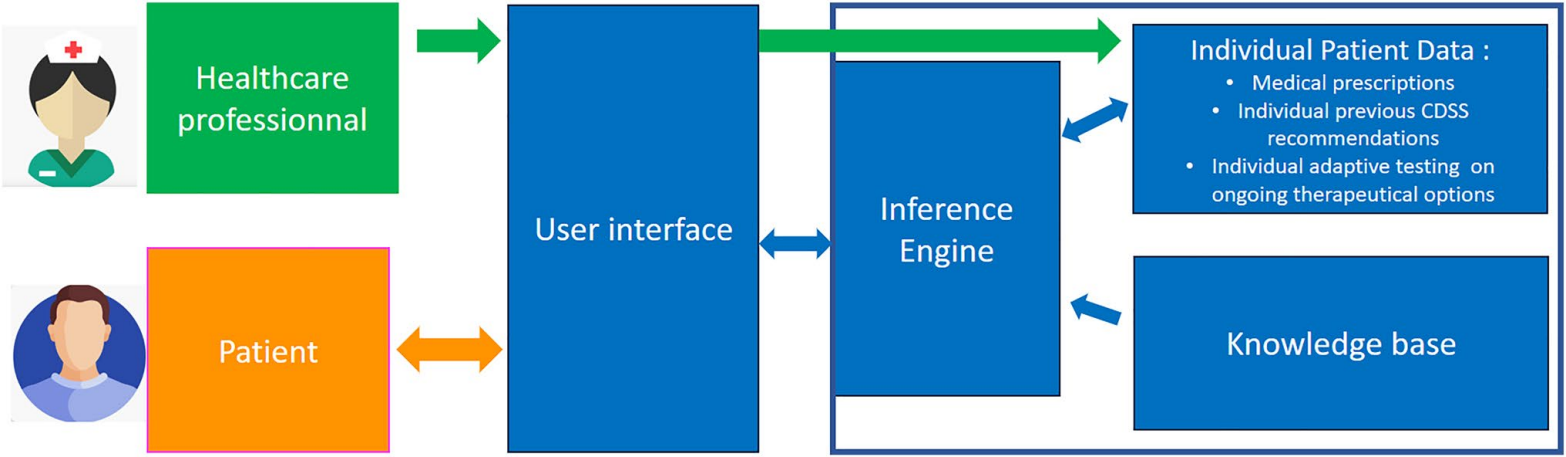
Diagram of key interactions in the experimental knowledge-base, patient clinical decision-support system

The experimental device is a knowledge-based, single system CDSS. Key interactions in this CDSS are presented in Fig. 1. Briefly, it is composed of an algorithmic base programmed into the system to model the decision combined with individual patient clinical data to generate an inference engine, and a communication interface. The algorithmic base is proprietary and therefore not shown. The knowledge base was built on medical knowledge from experts and medical literature in the form of a heuristic “if then” ruling and not on probabilistic inference [14, 15]. Individual patient clinical data related to pain, PONV and constipations prescriptions are integrated directly in the algorithmic program to generate an inference engine. Both knowledge base and individual patient data are built using Javascript Object Notation data exchange format using an ionic© software development kit. The user interface was designed to specifically fit in a smartphone display while optimizing patient understanding (Supplementary Fig. 1) and developed using Angular© framework. At each use of the device the individual patient data part is enriched by patient adaptive testing concerning the symptom treatment situation. For example, the device enquires about the symptom related pharmacological treatment adherence. After adaptive testing, the inference engine will then produce a symptom treatment recommendation and displays it to the patient (e.g. non opioid pharmacological therapy, non-pharmacological pain, PONV or ileus therapy, opioid, pharmacological PONV or ileus therapy). If the inference mechanism cannot provide a recommendation (e.g., every treatment option is already ongoing or in a lockout period) then an alarm message is generated to be emailed to the patient’s healthcare professional. The inference engine also considers potential interactions between symptom treatments and other symptoms and, if required, can produce adaptive testing and recommendations. For example, if the patient experiences PONV, and the device has recently recommended opioid medication for pain control, then the CDSS will recommend the addition of anti-nausea medication and continue to test for and recommend adherence to an opioid-sparing pain control strategy.

### Intervention

**Fig. 2:**
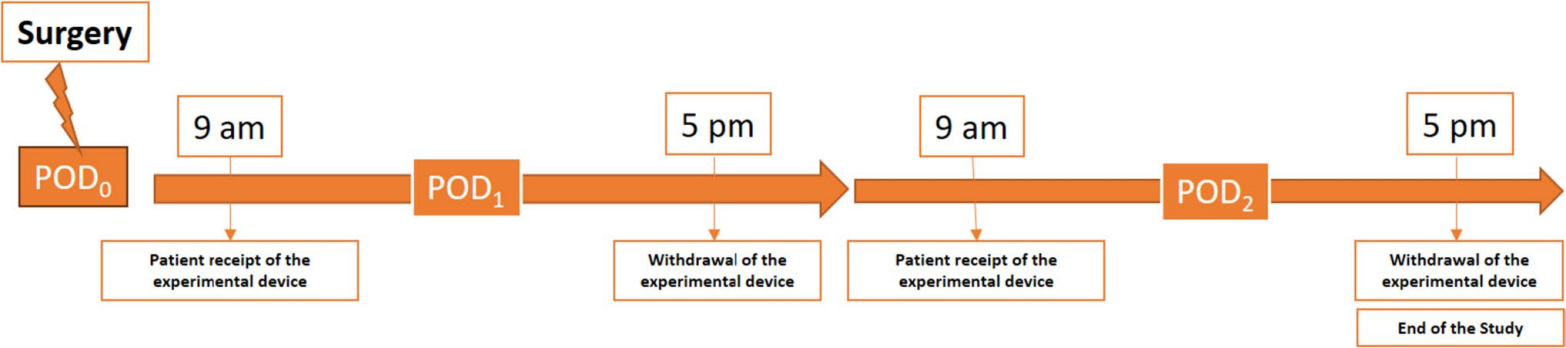
Experimental timeline schéma. *POD* post operative day

All study participants received the same intervention, i.e. use of our novel CDSS device, in this single-arm study. Before the intervention they received education from study personnel about the purpose and the use of the experimental device. After the surgical procedure, patients were given the experimental device on postoperative days 1 and 2 from 9 am to 5 pm. They were also given another educational session about the use of the device. The device was configured each day by study personnel before being handed to the patient. The configuration included medical prescriptions related to pain, PONV and constipation. The patients were instructed to press the room call button each time they experienced significant symptoms of pain, PONV or constipation to request help from the hospital team providing their routine care. In the educational session, they were instructed to activate the experimental device and go through the questions displayed, after they pressed the room call button. The patients and the hospital team were asked to disregard the recommendation provided by the experimental device recommendation. Each time a patient used the experimental device an automated email was immediately generated and sent by the device to the research team to inform them about the device activation. Sixty minutes after the device activation, study personnel visited the patient. At this time, they interpreted the same algorithm used by the device to determine the optimal medical response that the experimental CDSS should have produced in the particular patient situation, blinded from the real device recommendation. To establish this optimal response, the study personnel considered each time the medical prescriptions, the experimental rules of hierarchizations programmed into the CDSS and to the actual patient status (i.e. treatment already applied). The study personnel also interviewed the patient concerning any perceived time delay between routine call button activation and routine care delivery, satisfaction regarding the efficacy of routine care treatment, and the experimental device use. At the end of the last experimental day (i.e., postoperative day 2), the experimental team extracted the interaction history log from the device and compared the agreement between the recommendation provided by the experimental device and the recommendation provided by the blinded study personnel for each activation (Fig. 2).

As part of our enhanced recovery pathway, the oral route was the preferred route for analgesic administration after post-anesthesia care unit discharge.

### Outcomes

Primary and secondary outcomes were defined prior to initiation of the study.

The primary outcome analysis was the agreement between the recommendation provided by the experimental device and the recommendation provided by study personnel. The study personnel providing the recommendation (control) was blinded from the recommendation provided by the device. We pragmatically considered that a 90% threshold agreement would be clinically relevant.

Secondary outcomes included the time delay perceived by the patient between call button activation and routine care delivery, patient satisfaction regarding the efficacy of routine care treatment, and patient satisfaction regarding the experimental device use (from 0 no satisfaction to 100 absolute satisfaction). A secondary safety analysis was also performed based on the National Council for Medication Error Reporting and Prevention (NCC MERP) adapted scale (Level 1: no potential harm, level 2 monitoring or intervention potentially required to preclude harm, level 3: potential harm) [16, 17]. These assessments of potential harm were determined by a senior pharmaceutical doctor (co-author ARS). Oral Morphine Equivalent (OME) during hospital stay were calculated from postanesthesia care unit discharge until postoperative day 2 midnight [18].

## Sample size

As the primary outcome precluded a formal sample size calculation, we determined the required number of patients on a pragmatic basis for this pilot feasibility study. The target sample size was 30 patients who used the device at least once, in agreement with existing literature [19].

## Statistical methods

As no formal comparison tests were planned in this pilot, feasibility study, only descriptive statistical methods were used including percentage for categorical variables and median and quartiles for continuous variables. Bayesian methods were used to estimate the agreement level defined as the proportion of recommendations given by the device that were identical to the recommendation provided by study personnel (considered as the control, gold standard). The 95% credibility interval for agreement was calculated using a minimally informative Jeffreys Beta (0.5, 0.5) prior distribution. Each device use was considered independently. The statistical unit is thus each use of the device and not the patient. Statistical analyses were done with R v.4.2.2. (R Foundation, Austria).

## Results

**Fig. 3:**
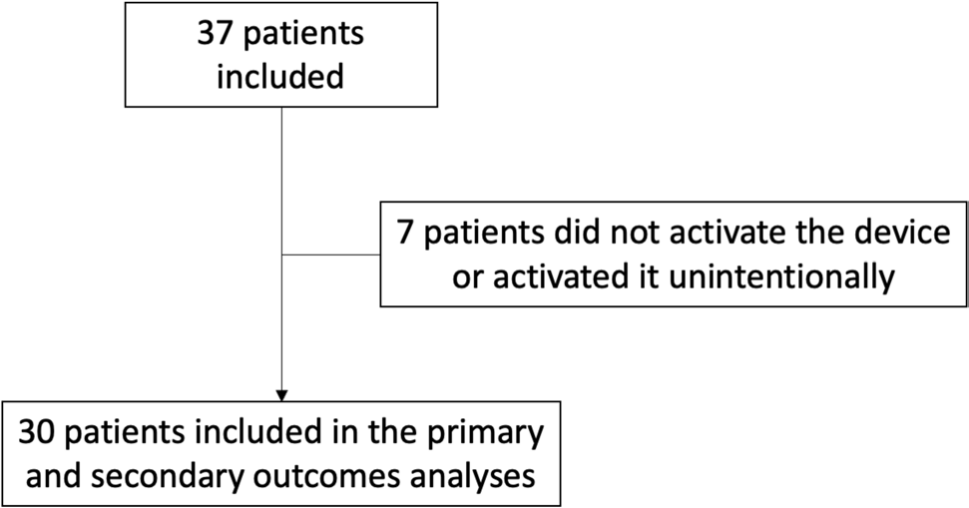
Experimental patient flow diagram. Experimental Fig. 1: Screenshot of patient interface display “home-page”. Experimental Fig. 2: Screenshot of an example of a recommendation on the experimental interface

The patients’ flowchart diagram is represented in Fig. 3. Overall, 37 patients were consented to accrue our target sample size of 30 (81%) patients who used the experimental device at least once.

The patients’ baseline demographics are reported in Table 1. The median (IQR) patient age was 64 (59–71) years old. Most patients (68%) were ASA II physical status. The most common surgical procedures were total hip arthroplasty (27%) and total knee arthroplasty (57%). Forty three percent of patients underwent surgery with general anesthesia and 57% with spinal anesthesia. Anesthesia was supplemented in 95% of the patients with a peripheral regional anesthesia.

**Table 1:** Baseline patients demographics

	Study cohort, n = 37
Age (year), median (IQR)	64 (59–71)
Ratio men/women, n	19/18 (1.1)
BMI, median (quartiles)	30,6 (28.1–33.7)
ASA physical status	
ASA I	3 (8%)
ASA II	25 (68%)
ASA III	9 (24%)
ASA IV	0 (0%)
Type of surgery	
THA	10 (27%)
TKA	21 (57%)
Revision THA	1 (3%)
Revision TKA	1 (3%)
TSA	0 (0%)
Spine	2 (5%)
Other	2 (5%)
Type of anesthesia	
GA	16 (43%)
Spinal anesthesia	21 (57%)
Supplementary regional anesthesia	35 (95%)
Hospital length of stay (days) mean, (SD)	6.7 (3.8)
OME, mg, median (SD)	26 (29)

*BMI* body mass index, *aSA* American Society of Anesthesiology, *THA* total hip arthroplasty, *TKA* total knee arthroplasty, *TSA* total shoulder arthroplasty, *GA* general anesthesia, *IQR* InterQuartil range, *SD* standard deviation, *OME* oral morphine equivalent (from PACU Discharge until postoperative day 2 midnight)

During the study period we observed 54 device uses, i.e. activations (Table 2) corresponding to 1.5 activations per patient. The main activation symptom was pain (46/54, 85%), then constipation (5/54, 9%), and PONV (3/54, 6%). The mean time delay between device activation and first use was 264 (+ 186) min.

**Table 2:** Experimental device activation

Device activations, n	54
Device activations per patient, n	1.5
Device activation for pain	46/54 (85%)
Device activation for PONV	3/54 (6%)
Device activation for constipation	5/54 (9%)

Concerning the primary outcome, an agreement between the treatment recommendation recommended by the experimental device and the research team was observed in 51 out of 54 activations (94.2%; 95%CI [85.9–98.4%]). The agreement level had a 86.6% probability to exceed the 90% clinically relevant agreement threshold. On 3 times there was no agreement. These 3 situations involved pain related activations. The severity of the every 3 unmatched uses was classified as level 1 (no potential harm). In one case of pain-related activation, the device recommended an opioid treatment but the research nurse recommended cryotherapy. The patient had falsely responded “yes” to the device asking if he add already undergone cryotherapy. In a second case of pain-related activation, the device recommended to call a healthcare professional for advice but the research nurse recommended an opioid treatment. The device had previously recommended an opioid treatment that the patient did not receive however, the device was considering the patient to be in the lockdown period for rescue opioid. In a third case of pain-related activation the device recommended an opioid treatment but the research nurse recommended non-opioid pain medication treatment. The patient had falsely responded “yes” to the device asking if he add already taken his non-opioid pain medication.

Agreement for all pain activations was 43/46 (93.2% [83.6–98.1%]) as shown in Table 3. Secondary outcomes are presented in Supplementary Table 1. The time delay perceived by the patient between the routine call button activation and routine care delivery was 5 (5–10) min. The global satisfaction rating concerning the use of the experimental device was 99% (90–100%). The global satisfaction rating regarding the efficacy of routine care treatment was 70% [50–80%].

**Table 3:** Agreement between experimental device and study personnel recommendations/*PONV* postoperative nausea and vomiting

	Pain	PONV	Constipation	Total
Agreement	43 (93%)	3 (100%)	5 (100%)	51 (94%)
No agreement	3 (7%)	0	0	3 (6%)
Total	46	3	5	54

## Discussion

Our study mainly demonstrates the feasibility of our knowledge-based, patient-centered, CDSS in a perioperative clinical setting. Perioperative care is characterized by the introduction of multiple symptom-specific treatments for pain, PONV and constipation in a period of patients’ physical and psychological vulnerability [6, 20, 21]. These symptom-specific treatments are multimodal and frequently include systemic and “on-demand”, pharmacological and non-pharmacological therapies [6, 21]. Moreover, some of these symptom-specific therapies may simultaneously ease some symptoms and trigger clinical adverse events. For example, opioids, which may be part of pain treatment protocol may induce PONV and constipation as a side effect [21].

A patient may also change location and caregivers during this perioperative period.

This challenging environment may contribute to suboptimal pain control despite significant progress that has been made in therapeutic solutions [1, 2]. Enhancing a patient’s adherence to the prescribed medical strategy wherever the location may therefore improve pain control and reduce opioid misuse. The same enhancement could also be expected for PONV and constipation management. We believe that patient CDSS are potential solutions to enhance patient adherence to the prescribed medical strategy. They also offer the potential, in combination with a secured pill delivery system, to autonomize patients in treating their own perioperative symptoms. This autonomy may improve the patient experience by reducing treatment delay and alleviate healthcare professional workload. Our study results suggest that there is a perceived time delay between the symptom demand and initiation of routine therapy. We also observed that patient satisfaction with the current routine therapy is often suboptimal.

Our study has several limitations. First being only single center, our results may have been different in another clinical setting. However, we believe that our university center perioperative care reflects many others worldwide. Second, as we only allowed participants to interact with the experimental device during daytime for study team members’ availability reasons, we may have observed different results during nighttime when routine clinicians are less available. Of note, we believe that during nighttime, the delay in routine therapeutic response may have been longer. Third, it is possible that routine clinicians being aware of the ongoing study may have made them particularly diligent in the response to patient’s symptoms (Hawthorne effect). Concerning the generalizability of our study results, they may differ depending on the patient profile and clinical setting. We excluded patients with neuropsychiatric or sensory disorders that may interfere with their use of the visual interface, therefore such patients may be less likely to benefit from the use of the experimental device. Fourth, the severity grading of the mismatch between the experimental device and the research team is subjective. For this reason we added the description of these mismatches in the results section. While not the focus of this study, we believe there will likely be value integrating data from our CDSS into the electronical health record data. This might improve healthcare professionals’ workload and patient monitoring efficacy.

According to available peer-reviewed literature, our study reports the first clinical assessment of a perioperative knowledge based CDSS. We hope that more research will add to this work to improve the care for patients in the perioperative period.

## Conclusion

The knowledge-based, patient CDSS we developed appears to have feasibility to provide appropriate treatment recommendations for pain, PONV and constipation in a perioperative clinical setting. More research is needed regarding further development of this patient CDSS and its potential benefits.

### Supplementary Information

Below is the link to the electronic supplementary material.

Supplementary file1 (DOCX 190 kb)

## Data Availability

The datasets used and/or analyzed during the current study are available from the corresponding author on reasonable request.

## Code availability

The underlying code for this study [and training/validation datasets] is not publicly available for proprietary reasons.
