# Supplementary material for: Knowledge-based, computerized, patient clinical decision support system for perioperative pain, nausea and constipation management: a clinical feasibility study
Source: J Clin Monit Comput. 2024 Apr 12;38(4):907–13. doi: 10.1007/s10877-024-01148-z (PMC11297814; doi:10.1007/s10877-024-01148-z)

| **Agreement between device and study personnel recommendation for PAIN, n(%)** | 43/46 (93%) |
| --- | --- |
| **Agreement between device and study personnel recommendation for PONV, n(%)** | 3/3 (100%) |
| **Agreement between device and study personnel recommendation for CONSTIPATION, n(%)** | 5/5 (100%) |
|  |  |
| **Time delay perceived by the patient between call button activation and routine care delivery for PAIN, min, median (quartiles)** | 5 (5 - 10) |
| **Time delay perceived by the patient between call button activation and routine care delivery for PONV, min, median (quartiles)** | 15 (8 - 37) |
| **Time delay perceived by the patient between call button activation and routine care delivery for CONSTIPATION, min, median (quartiles)** | 9 (6 - 12) |
|  |  |
| **Satisfaction concerning the use of experimental device for PAIN, % maximal satisfaction, median (quartiles)** | 100 (90 - 100) |
| **Satisfaction concerning the use of experimental device for PONV, % maximal satisfaction, median (quartiles)** | 100 (95 - 100) |
| **Satisfaction concerning the use of experimental device for CONSTIPATION, % maximal satisfaction, median (quartiles)** | 90 (90 - 95) |
|  |  |
| **Satisfaction regarding the efficacy of routine care treatment for PAIN, % maximal satisfaction, median (quartiles)** | 70 (50 - 80) |
| **Satisfaction regarding the efficacy of routine care treatment for PONV, % maximal satisfaction, median (quartiles)** | 60 (40 - 75) |
| **Satisfaction regarding the efficacy of routine care treatment for CONSTIPATION, % maximal satisfaction, median (quartiles)** | 50 (46 - 62) |

Supplemental Table 1: secondary outcomes

Supplementals Figures

Supplemental Figure 1 : examples of patient interface display


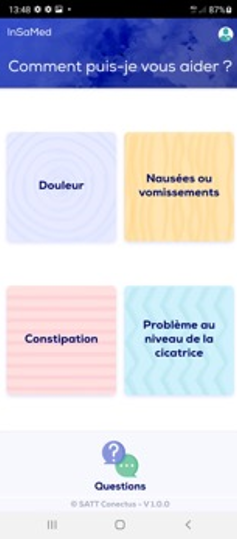


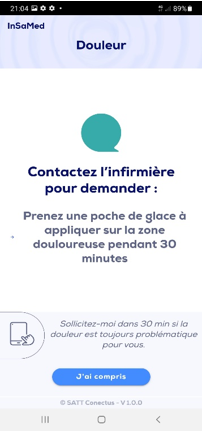

Supplement: Supplementary file 1 — Supplementary file1 (DOCX 190 kb) [file 10877_2024_1148_MOESM1_ESM.docx]
